# Supplementary material for: Nutritional Status and Quality of Life in Hospitalised Cancer Patients Who Develop Intestinal Failure and Require Parenteral Nutrition: An Observational Study
Source: Nutrients. 2020 Aug 7;12(8):2357. doi: 10.3390/nu12082357 (PMC7468734; doi:10.3390/nu12082357)
Supplement: Supplementary file 1 [file nutrients-12-02357-s001.pdf]

Supplementary Materials

1. Supplementary Tables

Table S1. Data collection timeline

Parameter	From patient records (closest measurement to referral)	From dietitian during ward round
Demographics	X	
Medical information	X	
Serum biochemistry	X	
Anthropometric data		X
Malnutrition screening	X	
Quality of Life		X
Performance status		X

Table S2. UCLH Nutrition Screening Tool

UCLH Nutrition Screening Tool
All patients to be weighed and screened within 24 hours of admission then weekly.
Please circle a score for each category in the relevant column.

	**Adm**	**Wk 1**	**Wk 2**	**Wk3**	**Wk4**	**Wk5**	**Wk6**	**Wk7**
**Date:**<br>**Time:**								
**Weight: (please state reason if unable to weigh patient by either scales and hoist)**								
**Height:**								
**BMI:**								
**Appetite**								
Normal for patient	0	0	0	0	0	0	0	0
Reduced	3	3	3	3	3	3	3	3
Minimal	5	5	5	5	5	5	5	5
**Neurological / psychological status**								
Fully orientated	0	0	0	0	0	0	0	0
Confused	3	3	3	3	3	3	3	3
Unconscious	5	5	5	5	5	5	5	5
**Physical Appearance**								
Appears appropriate weight or overweight	0	0	0	0	0	0	0	0
Appears thin	2	2	2	2	2	2	2	2
Appears emaciated	4	4	4	4	4	4	4	4
**Weight Loss**								
No weight loss	0	0	0	0	0	0	0	0
Gradual over several months	2	2	2	2	2	2	2	2
Rapid over several weeks	5	5	5	5	5	5	5	5
Unable to score	2	2	2	2	2	2	2	2
**Intake**								
Manages most of 3 meals a day or equivalent	0	0	0	0	0	0	0	0
Poor intake leaving > half meals offered	3	3	3	3	3	3	3	3
Receiving parenteral or enteral nutrition	3	3	3	3	3	3	3	3
**Total score:**								
**Sign & print name:**								
**Referred to dietitian: Yes/No (sign)**								

Please see reverse for appropriate actions and create an individualised patient care plan

**Actions**	
**Score 0-2**<br>**Low risk**	**ROUTINE CLINICAL CARE**<br>**Referral to the dietitian is not required**<br><br>• Weigh and screen patient weekly and document<br>• Ensure that help and advice on appropriate menu choices is provided should the patient require a Kosher, Halal, Caribbean, vegetarian, soft or puree diet
**Score 3 – 6**<br>**Medium risk**	**OBSERVE**<br>**Referral to the dietitian is not required**<br><br>• Weigh and screen patient weekly and document.<br>• **For any patients on parenteral or enteral nutrition, please ensure that the dietitian is informed**<br>• Check that any symptoms affecting food intake are adequately controlled, e.g. nausea, vomiting, constipation or abdominal pain<br>• Check patient:<br>o Can reach food/feed self /use utensils/cut up food<br>o Can chew (check dentures)/swallow<br>o Has adequate mouth care<br>o Is positioned correctly for eating and drinking<br>• Encourage patient to choose ☺ foods from menu.<br>• If assistance with feeding is required inform catering staff of need for **red tray/ red jug.**<br>• Offer Full Fat Milk/ soups between meals.<br>• Offer the Snack Menu where available for more choice.<br>• Commence food record charts.
**Score 7 or more**<br>**High risk**	**TREAT**<br>**Refer to dietitian**<br><br>• **Refer to the dietitian and commence food record charts for 2-3 days**<br>• Encourage patient to choose ☺ foods from menu.<br>• If assistance with feeding is required inform catering staff of need for red tray/ red jug.<br>• Offer Full Fat Milk/ soups between meals.<br>• Offer the Snack Menu where available for more choice.

Table S3. Agreement among categorical variables of nutritional status with Cohen's kappa.

Variable	Kappa	P-value
**PG-SGA categories**		
NST categories	-0.147	0.292
Weight loss% categories	0.022	0.820
BMI categories	-0.027	0.898
**NST categories**		
Weight loss % categories	0.007	0.929
BMI categories	-0.090	0.510

BMI = Body Mass Index, Kappa=Cohen's Kappa agreement coefficient, NST = Nutritional screening tool, PG-SGA = Patient Generated Subjective Global Assessment

Table S4. Spearman correlations between variables. Only correlations with p<0.05 are presented.

Variable 1	Variable 2	Spearman's rho	p-value	N
**KPS Score**	Length of stay	-0.448	0.015	29
**NST score**	MUAMC	-0.426	0.043	23
**NST score**	Weight on admission	-0.455	0.022	25
**NST score**	Weight loss in 6months (%)	0.502	0.015	23
**TSF**	MUAMC	0.727	0.000	27
**TSF**	BMI on admission	0.531	0.005	26
**TSF**	Weight on admission	0.469	0.014	27
**TSF**	Weight loss in 6months (%)	0.425	0.034	25
**MAC**	BMI on admission	0.553	0.003	26
**MAC**	Weight on admission	0.683	0.000	27
**MAC**	MUAMC	0.770	0.000	27
**MUAMC**	BMI on admission	0.805	0.000	26
**MUAMC**	Weight on admission	0.830	0.000	27
**MUAMC**	Weight loss in 6months	0.475	0.017	25
**MUAMC**	Weight loss in 6months (%)	0.580	0.002	25
**HGS**	Weight on admission	0.386	0.047	27
**HGS**	MAC	0.440	0.022	27
**HGS**	KPS	0.531	0.004	27
**Energy/independence**	Age	0.525	0.018	20
**Pain**	HGS	-0.474	0.040	19

BMI = Body Mass Index, HGS = hand grip strength, KPS = Karnofsky Performance Status, MAC = mid arm circumference, MUAMC = mid upper arm muscle circumference, NST = Nutritional screening tool, TSF = tricep skinfold thickness.

Table S5. Cancer cachexia distribution according to type of malignancy, KPS score, metastatic disease, and indication for PN.

	Cancer Cachexia [N(%)]		χ^2 p-value
	No	Yes	
**Type of malignancy**			
Gynaecological	0 (0.0)	2 (11.1)	0.082
Upper Gastrointestinal	2(25.0)	7 (38.9)	
Lower Gastrointestinal	0 (0.0)	4 (22.2)	
Haematological	4 (50.0)	5 (27.8)	
Other	2 (25.0)	0 (0.0)	
**KPS**			
<50	3 (37.5)	4 (22.2)	0.418
≥50	5 (62.5)	14 (77.8)	
**Metastasis**			
No	6 (75.0)	8 (38.9)	0.089
Yes	2 (25.0)	11 (61.1)	
**Indication for HPN**			
Extensive small bowel mucosal disease	4 (44.4)	8 (38.1)	0.671
Intestinal dysmotility	2 (22.2)	2 (9.5)	
Mechanical obstruction	3 (33.3)	10 (47.6)	
Short bowel syndrome or intestinal fistula	0 (0.0)	1 (4.8)	

Gynaecological: breast, endometrial; Lower gastrointestinal: small bowel, colon, sigmoid; upper gastrointestinal: oesophageal, gastric; haematological: leukaemia, lymphoma, amyloidosis; other: penis, bladder, HPN=Home Parenteral Nutrition, KPS=Karnofsky Performance Status.

Table S6. Metastatic disease distribution according to type of malignancy, KPS score, and indication for PN.

	Metastatic Disease [N (%)]		χ^2 p- value
	No	Yes	
**Type of malignancy**			
Gynaecological	0 (0.0)	2 (14.3)	0.021
Upper Gastrointestinal	5 (31.3)	6 (42.9)	
Lower Gastrointestinal	0 (0.0)	4 (28.6)	
Haematological	10 (62.5)	1 (7.1)	
Other	1 (6.3)	1 (7.1)	
**KPS**			
<50	7 (43.8)	2 (14.3)	0.084
≥50	9 (56.3)	12 (85.7)	
**Indication for HPN**			
Extensive small bowel mucosal disease	11 (68.8)	1 (7.1)	**0.001**
Intestinal dysmotility	3 (18.8)	1 (7.1)	
Mechanical obstruction	2 (12.5)	11 (78.6)	
Short bowel syndrome or intestinal fistula	0 (0.0)	1 (7.1)	

Gynaecological: breast, endometrial; Lower gastrointestinal: small bowel, colon, sigmoid; upper gastrointestinal: oesophageal, gastric; haematological: leukaemia, lymphoma, amyloidosis; other: penis, bladder, HPN=Home Parenteral Nutrition, KPS=Karnofsky Performance Status.

Table S7. NST score distribution according to type of malignancy, KPS score, cachexia, metastatic disease, and indication for PN.

	NST scores [N (%)]			χ^2 p- value
	**Low risk**	**Medium Risk**	**High Risk**	
**Type of malignancy**				
Gynaecological	0 (0.0)	1 (25.0)	1 (6.3)	0.623
Upper Gastrointestinal	1 (20.0)	1 (25.0)	6 (37.5)	
Lower Gastrointestinal	0 (0.0)	0 (0.0)	3 (18.8)	
Haematological	3 (60.0)	2 (50.0)	5 (31.3)	
Other	1 (20.0)	0 (0.0)	1 (6.3)	
**KPS**				
<50	1 (20.0)	1 (25.0)	3 (18.8)	0.962
≥50	4 (80.0)	3 (75.0)	13 (81.3)	
**Cancer Cachexia**				
No	3 (60.0)	2 (50.0)	2 (14.3)	0.105
Yes	3 (40.0)	2 (50.0)	12 (85.7)	
**Metastasis**				
No	4 (80.0)	2 (50.0)	7 (43.8)	0.365
Yes	1 (20.0)	2 (50.0)	9 (56.3)	
**Indication for HPN**				
Extensive small bowel mucosal disease	4 (80.0)	1 (25.0)	5 (31.3)	0.483
Intestinal dysmotility	0 (0.0)	0 (0.0)	2 (12.5)	
Mechanical obstruction	1 (20.0)	3 (75.0)	8 (50.0)	
Short bowel syndrome or intestinal fistula	0 (0.0)	0 (0.0)	1 (6.3)	

Gynaecological: breast, endometrial; Lower gastrointestinal: small bowel, colon, sigmoid; upper gastrointestinal: oesophageal, gastric; haematological: leukaemia, lymphoma, amyloidosis; other: penis, bladder, HPN=Home Parenteral Nutrition, KPS=Karnofsky Performance Status, NST=Nutritional Screening Tool.

Table S8. PG-SGA score distribution according to type of malignancy, KPS score, cachexia, metastatic disease, and indication for PN.

	**PG-SGA scores [N (%)]**		**χ^2 p- value**
	**Medium Risk**	**High Risk**	
**Type of malignancy**			
Gynaecological	0 (0.0)	1 (5.9)	0.909
Upper Gastrointestinal	2 (28.6)	6 (35.3)	
Lower Gastrointestinal	1 (14.3)	3 (17.6)	
Haematological	3 (42.9)	6 (35.3)	
Other	1 (14.3)	1 (5.9)	
**KPS**			
<50	2 (28.6)	4 (23.5)	0.795
≥50	5 (71.4)	13 (76.5)	
**Cancer Cachexia**			
No	2 (28.6)	5 (33.3)	0.823
Yes	5 (71.4)	10 (66.7)	
**Metastasis**			
No	4 (57.1)	9 (52.9)	0.851
Yes	3 (42.9)	8 (47.1)	
**Indication for HPN**			
Extensive small bowel mucosal disease	2 (28.6)	6 (35.3)	0.467
Intestinal dysmotility	1 (14.3)	3 (17.6)	
Mechanical obstruction	3 (49.2)	8 (47.1)	
Short bowel syndrome or intestinal fistula	1 (14.3)	0 (0.0)	

Gynaecological: breast, endometrial; Lower gastrointestinal: small bowel, colon, sigmoid; upper gastrointestinal: oesophageal, gastric; haematological: leukaemia, lymphoma, amyloidosis; other: penis, bladder, HPN=Home Parenteral Nutrition, KPS=Karnofsky Performance Status, PG-SGA=Patient Generated Subjective Global Assessment.

Table S9. Factor analysis and structure.

	Questions	Factors				
		1	2	3	4	5
**Pain**	Have you had pain after eating or drinking?	-0.912				
	Have you had other pain?	-0.875				
	Have you had aches or pains in your muscles or joints?	-0.749				
	Were you able to eat food?	-0.743				
	Have you had nausea/vomiting?	-0.702				
	Have you had trouble taking a short walk outside the house?	-0.685				
**Worrying**	Have you worried about the future?		-0.897			
	Have you worried about your current health?		-0.848			
	Have you felt bloated?		-0.612			
**Walking/ socialising**	Have you had trouble taking a long walk?			0.852		
	Were you able to go out to work?			0.684		
	Have you felt you wanted to go out to work?			0.602		
	Were you able to socialise?			0.578		
	Have you needed help with eating, dressing, washing yourself or using the toilet?			0.547		
**Energy/ independence**	Were you able to feel independent?				0.853	
	Have you felt depressed?				0.674	
	Were you able to cope with daily life?				0.647	
	Were you able to drink fluids?				0.625	
	Have you felt lacking in energy?				0.622	
	Have you felt tired?				0.554	
**Activities**	Have you had trouble doing strenuous activities, like carrying a heavy shopping bag or suitcase?					0.697
	Were you able to take part in hobbies or leisure activities?					0.688
	Were you able to do shopping?					0.666
	Were you able to exercise?					0.638

Table S10. Quality of Life according to type of malignancy, indication for PN, presence of metastases and cachexia, NST, PG-SGA, KPS and WHO-PS scores.

	FACT-G	FACT-G PHYSICAL	FACT-G SOCIAL	FACT-G EMOTIONAL	FACT-G FUNCTIONAL	Pain	Worrying	Walking/ Socialising	Energy/ Independence	Activities
**Type of malignancy**										
Gynaecological	30.0	10.0	24.0	24.0	6.0	1.8	-0.6	0.8	-0.5	-0.9
Upper Gastrointestinal	26.0 (8.4)	9.35 (3.6)	21.9 (7.2)	12.7 (5.8)	12.3 (6.7)	0.3 (0.5)	0.2 (1.1)	-0.2 (0.7)	0.0 (0.8)	0.3 (1.3)
Lower Gastrointestinal	32.1 (11.7)	13.7 (5.7)	25.1 (1.9)	16.3 (2.5)	13.5 (8.2)	-0.2 (1.0)	-0.2 (0.7)	0.2 (0.6)	0.0 (1.4)	0.7 (0.8)
Haematological	24.0 (11.1)	11.1 (7.2)	24.7 (3.0)	14.9 (7.7)	8.3 (6.2)	-0.3 (1.2)	0.1 (0.8)	0.3 (1.4)	-0.2 (1.1)	-0.3 (0.8)
Other	27.7 (13.7)	12.5 (12.0)	14.3 (6.1)	14.5 (6.4)	10.7 (6.6)	-0.6 (0.8)	-0.4 (2.3)	-0.9 (0.4)	0.8 (1.5)	-0.4 (0.2)
p-value	0.766	0.806	0.169	0.514	0.614	0.210	0.893	0.496	0.821	0.422
**Metastatic Disease**										
No	26.9 (11.0)	12.9 (5.7)	23.7 (4.4)	16.2 (6.4)	10.0 (6.8)	-0.3 (0.9)	-0.2 (1.2)	0.5 (1.2)	0.1 (1.2)	-0.2 (0.9)
Yes	26.4 (8.9)	9.0 (5.5)	22.0 (6.8)	13.2 (5.8)	11.6 (6.5)	0.3 (1.1)	0.2 (0.8)	-0.4 (0.7)	-0.1 (0.8)	0.2 (1.1)
p-value	0.900	0.103	0.477	0.249	0.561	0.197	0.486	0.835	0.527	0.416
**Cancer Cachexia**										
No	32.2 (10.0)	14.3 (8.1)	20.6 (6.1)	17.4 (4.3)	13.3 (6.7)	-0.5 (1.0)	-0.1 (1.5)	0.3 (1.3)	0.2 (1.1)	-0.6 (0.2)
Yes	24.9 (9.8)	10.2 (4.1)	24.0 (5.4)	14.0 (6.3)	9.8 (6.9)	0.1 (0.9)	0.1 (0.8)	-0.1 (0.9)	0.0 (1.0)	0.2 (1.1)
p-value	0.136	0.130	0.219	0.249	0.316	0.204	0.672	0.445	0.769	0.134
**NST**										
Low risk	25.4 (7.1)	10.0 (6.0)	22.2 (7.4)	14.4 (7.8)	9.8 (3.6)	-0.5 (0.9)	0.9 (0.9)	0.7 (1.5)	0.1 (0.4)	0.0 (0.7)
Medium risk	28.0 (18.4)	14.7 (12.2)	25.4 (2.0)	17.0 (7.1)	11.0 (11.3)	-1.1	-1.3	0.3	-1.2	-0.9
High risk	28.1 (10.4)	10.7 (5.6)	23.3 (5.7)	14.4 (6.5)	12.1 (7.2)	0.1 (1.1)	-0.1 (0.9)	-0.4 (0.8)	0.3 (1.1)	0.0 (1.2)
p-value	0.884	0.667	0.819	0.877	0.812	0.344	0.075	0.203	0.378	0.764
**PG-SGA**										
At risk	28.0 (11.1)	14.2 (6.8)	22.0 (7.3)	15.4 (6.2)	11.2 (6.6)	-0.6 (0.7)	-0.4 (1.1)	-0.4 (0.8)	0.6 (1.1)	0.2 (1.4)
Severely malnourished	25.8 (9.8)	9.4 (5.0)	23.5 (5.0)	14.8 (6.3)	9.9 (6.3)	0.2 (1.0)	0.2 (0.9)	0.1 (1.0)	-0.3 (0.8)	-0.1 (0.8)
p-value	0.643	0.075	0.590	0.827	0.673	0.078	0.181	0.265	0.053	0.508
**KPS**										
<50	20.6 (9.5)	9.3 (3.2)	19.7 (8.9)	11.9 (5.4)	7.7 (4.7)	-0.1 (1.0)	0.3 (0.9)	0.1 (1.4)	-0.3 (0.7)	-0.4 (0.6)
≥50	28.2 (9.5)	11.4 (6.3)	23.7 (4.4)	15.4 (6.3)	11.6 (6.9)	0.0 (1.0)	-0.1 (1.0)	0.0 (0.8)	0.1 (1.1)	0.2 (1.1)
p-value	0.127	0.475	0.164	0.268	0.254	0.855	0.433	0.750	0.354	0.284
**WHO-PS**										
1	34.7 (11.6)	10.7 (5.7)	25.5 (2.2)	18.0 (2.0)	14.4 (8.4)	0.5 (0.4)	-0.5 (0.8)	-0.6 (0.1)	-0.3 (1.6)	1.1 (1.9)
2	27.5 (9.6.)	11.7 (5.3)	24.3 (3.3)	15.5 (6.8)	11.4 (7.5)	0.0 (1.0)	0.0 (0.9)	0.2 (0.9)	0.0 (1.1)	0.2 (0.9)
3	22.8 (8.7)	10.1 (7.0)	20.1 (7.8)	12.5 (5.9)	8.7 (4.3)	-0.1 (1.1)	0.1 (1.2)	-0.1 (1.2)	0.1 (0.8)	-0.5 (0.5)
p-value	0.178	0.848	0.167	0.351	0.411	0.774	0.697	0.640	0.845	0.068

Gynaecological: breast, endometrial; Lower gastrointestinal: small bowel, colon, sigmoid; upper gastrointestinal: oesophageal, gastric; haematological: leukaemia, lymphoma, amyloidosis; other: penis, bladder. FACT-G=Functional Assessment of Cancer Therapy: General, NST=Nutritional Screening Tool, PG-SGA-=Patient Generated Subjective Global Assessment, KPS= Karnofsky Performance Status, WHO-PS=World Health Organisation Performance Status

2. Supplementary Figures

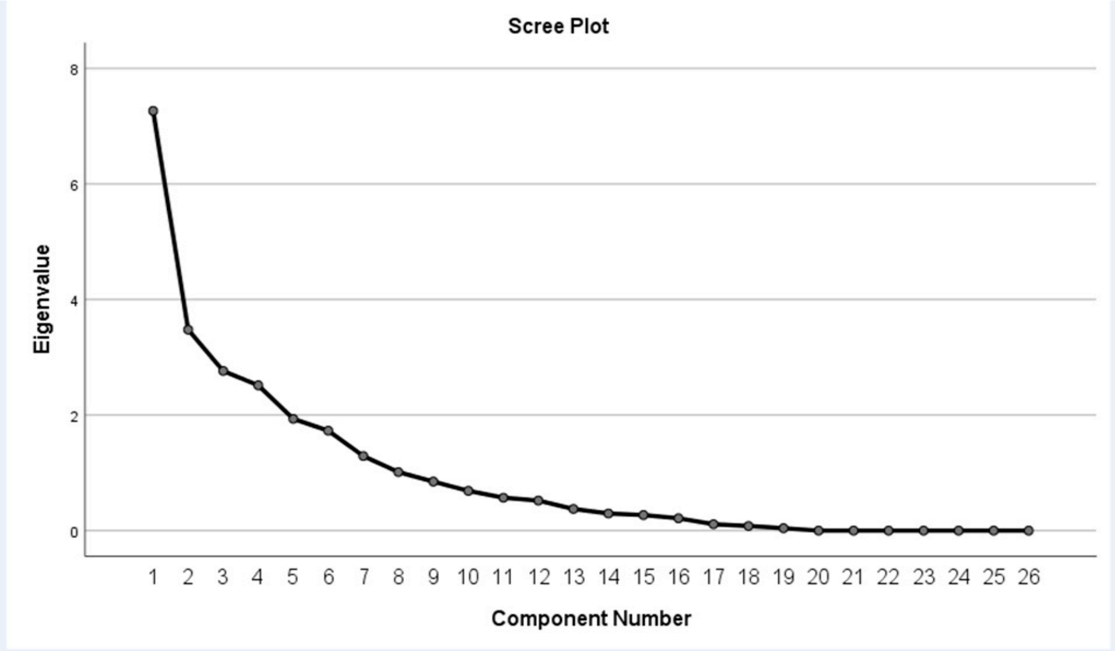

Figure S1. Scree plot. The components lie in the X-axis and the corresponding eigenvalues in the Y-axis. The line drop after the fifth component indicates that the factor analysis identified five factors.
